# Microarray Analysis of the Genomic Effect of Eugenol on Methicillin-Resistant *Staphylococcus aureus*

**DOI:** 10.3390/molecules27103249

**Published:** 2022-05-19

**Authors:** Ayuba Sunday Buru, Vasantha Kumari Neela, Kavitha Mohandas, Mallikarjuna Rao Pichika

**Affiliations:** 1 Department of Medical Laboratory Science, College of Medicine and Health Sciences, Afe Babalola University, Ado-Ekiti 360101, Ekiti State, Nigeria; 2 Department of Medical Microbiology and Parasitology, Faculty of Medicine and Health Sciences, Universiti Putra Malaysia, Serdang 43400, Malaysia; vasantha@upm.edu.my; 3 Department of Microbiology, Faculty of Medicine, MAHSA University College, Kuala Lumpur 59100, Malaysia; kmohandas@mahsa.edu.my; 4 Department of Medicinal Chemistry, International Medical University, No. 126, Jalan 19/155B, Bukit Jalil, Kuala Lumpur 57000, Malaysia; mallikarjunarao_pichika@imu.edu.my

**Keywords:** DNA microarray, gene transcription, eugenol, methicillin-resistant *Staphylococcus aureus* (MRSA)

## Abstract

*Staphylococcus aureus* is a highly adaptive human pathogen responsible for serious hospital- and community-acquired infectious diseases, ranging from skin and soft tissue infections, to complicated and life-threatening conditions such as endocarditis and toxic shock syndrome (TSS). The rapid development of resistance of this organism to available antibiotics over the last few decades has necessitated a constant search for more efficacious antibacterial agents. Eugenol (4-allyl-2-methoxyphenol) belongs to the class of chemical compounds called phenylpropanoids. It is a pure-to-pale yellow, oily liquid substance, mostly extracted as an essential oil from natural products such as clove, cinnamon, nutmeg, basil, and bay leaf. Eugenol has previously been shown to have antimicrobial activity against methicillin-resistant *Staphylococcus aureus* (MRSA). However, the mechanism of action of eugenol against MRSA has not, as yet, been elucidated; hence, the necessity of this study. Global gene expression patterns in response to challenge from subinhibitory concentrations of eugenol were analysed using the Agilent DNA microarray system to identify genes that can be used as drug targets—most importantly, essential genes involved in unique metabolic pathways elicited for bacterial survival. Transcriptomic analysis of fluctuating genes revealed those involved in amino acid metabolism, fatty acid metabolism, translational, and ribosomal pathways. In amino acid metabolism, for instance, the *argC* gene encodes for *N*-acetyl-gamma-glutamyl-phosphate reductase. The *argC* gene plays an important role in the biosynthesis of arginine from glutamate in the amino acid metabolic pathway. It is the enzyme that catalyses the third step in the latter reaction, and without this process the production of *N*-acetylglutamate 5-semialdehyde cannot be completed from the NADP-dependent reduction of *N*-acetyl-5-glutamyl phosphate, which is essential for the survival of some microorganisms and plants. This study enables us to examine complete global transcriptomic responses in MRSA when challenged with eugenol. It reveals novel information with the potential to further benefit the exploratory quest for novel targets against this pathogen, with a view to the development of efficacious antimicrobial agents for the treatment of associated infections.

## 1. Introduction

Methicillin-resistant *Staphylococcus aureus* (MRSA) infections and related clinical complications are on the increase. MRSA is a highly adaptive human pathogen causing benign skin and soft tissue infections, as well as life-threatening endocarditis and toxic shock syndrome (TSS) [1]. As a major nosocomial pathogen, it is implicated in both hospital- and community-acquired staphylococcal infections that are difficult to treat and control, due to a multifactorial combination of toxin-mediated virulence, invasiveness, and antibiotic resistance [2]. There has been a widespread increase in the resistance of *S. aureus* to antibiotics over the past decade, and this has posed a great challenge for the development of effective therapeutics against this opportunistic pathogen [2].

Antibiotic resistance in *Staphylococcus aureus* is associated with staphylococcal cassette chromosome *mec* (SCC*mec*), known to encode for the *mecA* gene, which confers resistance to methicillin and other beta-lactam antibiotics [1]. The occurrence of resistance within and across different antibacterial classes has reinforced the urgent need for the discovery of new and potent compounds aiming at novel cellular metabolic functions hitherto not targeted by current antibacterial agents [2].

According to a 2007 CDC report, the rate of mortality and morbidity due to MRSA in the U.S.A is on the increase. As such, the significance of this organism and its infections in relation to public health should not be underestimated [3]. Therefore, there is a significant need for more research into natural plant-based biodiversity to provide an alternative source of more efficacious antimicrobial secondary metabolites against this pathogen [2]. The current strategy for novel antibacterial discovery from natural products involves screening and expression analysis of promising targets against *S. aureus*. These targets encompass multifarious functionalities, including fatty acid metabolism, peptidoglycan and protein biosynthesis, cell division, DNA replication, and biosynthesis [4].

Plants and plant-derived natural products are sources of secondary metabolites such as terpenoids, pentacyclic triterpenoids, flavonoids, and glycosides that have shown promising anti-staphylococcal activities [2,5,6,7,8]. Eugenol, a phenylpropanoid, is found abundantly in the essential oils of species such as *Syzygium aromaticum*, *Cinnamomum zeylanicum*, *Myristica fragrans*, *Ocimum basilicum*, *Illicium verum*, *Melissa officinalis*, *Anethum graveolens*, and *Laurus nobilis* [9]. Eugenol is used in perfumes, flavourings, essential oils, and in medicines as a local antiseptic and anaesthetic. Previous studies have shown that eugenol has antimicrobial, antioxidant, anti-inflammatory, anti-carminative, and antispasmodic activities, and kills certain human colon cancer cell lines in vitro [10]. Similar studies on other plant-derived compounds showing anti-staphylococcal activities have been reported. In a preliminary study, betulinaldehyde has been reported to exhibit antibacterial activity against *S. aureus*, with an MIC value of 512 mg/mL. This compound potentiates the activity of the cell wall inhibitors methicillin, vancomycin [11], berberine chloride [12], cryptotanshinone [13], and rhein [14].

Therefore, this study had the objective of ascertaining the transcriptional profile of genes expressed due to exposure of MRSA to subinhibitory concentrations of eugenol, and of fully understanding its possible mechanisms of action. Additionally, the genes upregulated when challenged with eugenol may also be explored as potential novel drug targets for the development of other therapeutics that synergise with eugenol in combating MRSA infections. 

## 2. Results and Discussion

The microarray and RT-qPCR platforms have facilitated a better understanding of transcriptional changes due to treatment of MRSA with eugenol, as correlated with expression levels. To determine the genomic effect of eugenol on MRSA, we first exposed exponentially growing cells of MRSA to 0.0012 mg/mL eugenol (dissolved in 10% DMSO), and concurrently demonstrated growth inhibition of MRSA at 60 min. Table 1 and Table 2 below list some of the important metabolic pathways that fluctuated upon treatment of MRSA with eugenol. 

Table 3, Table 4 and Table 5 describe quality control data for the validation of microarray results with qPCR.

### 2.1. Gene Transcriptional Responses in MRSA upon Treatment with Eugenol

Treatment of MRSA with subinhibitory concentrations of eugenol revealed that a total of 13 genes had fluctuated—7 genes were upregulated whilst 6 were downregulated—in the transcriptional changes that had occurred, as shown in Table 1. Of the total 13 genes, 6 are hypothetical genes, 2 genes are involved in amino acid metabolism, 4 are involved in genetic information processing—i.e., ribosomal and translational biogenesis metabolism—and 1 is involved in carbohydrate metabolism, as shown in Table 2. With further validation using qPCR, two genes—*luxS* and *argC*—passed through the validation processes. These genes are involved in cysteine and methionine metabolism, and in arginine and proline metabolism, respectively.

### 2.2. Cysteine and Methionine Metabolism

The *luxS* gene encodes for *S*-ribosylhomocysteinase (*luxS*), which is also involved in the amino acid metabolic pathway. It was downregulated by 2.4–3.0-fold when treated with eugenol. *luxS*, as an Fe^2+^-dependent metalloenzyme, plays a catalytic role in *S*-ribosylhomocysteine (SRH)–thioether bond cleavage to produce homocysteine (Hcys) and 4,5-dihydroxy-2,3-pentanedione (DPD) [15]. The enzyme *S*-(5-deoxy-d-ribos-5-yl)-L-homocysteine l-homocysteine-lyase [(4*S*)-4,5-dihydroxypentan-2,3-dione-forming] belongs to the class of carbon–sulphur lyases and the family of lyases [16], and is also commonly referred to as *S*-ribosylhomocysteinase and *luxS* [17]. The enzymes are involved in methionine metabolism [17], which is a key step in the biosynthetic pathway of the type II autoinducer (AI-2), which plays a vital role in the quorum-sensing mechanisms of both Gram-positive and Gram-negative bacteria [18]. AI-2 functions as a signalling molecule in interspecies communication through regulation of niche-specific genes that have diverse functions in various bacterial species, which is often in response to a change in the population density [17]. This response serves a dual purpose, i.e., for detoxification via elimination/metabolism of *S*-adenosylhomocysteine, and also for the production of a type II quorum-sensing molecule [19]. Cell-population-density-dependent regulation of gene expression is a key contributing factor to bacterial pathogenesis. There are two quorum-sensing (QS) systems in the staphylococci [20], namely, the accessory gene regulator (*agr*), and *luxS*. In staphylococci, upregulation of *luxS* reduces biofilm formation and virulence rather than inducing it, especially during biofilm-associated infection. The *agr* gene has been shown to enhance biofilm detachment through upregulation of detergent-like peptides, whilst *luxS* decreases cell–cell adhesion through downregulation of biofilm exopolysaccharide expression [20]. The metabolically inert mode of growth of biofilms appears to be characterised somewhat by overall low activity of the staphylococcal QS systems [19]. In this study, when MRSA was challenged with eugenol, downregulation of *luxS* expression was indicative of heightened biofilm production and heightened virulence of the bacterium [19], which are of course the typical immediate self-defence responses against aggressive threats. As such, inducers of *luxS*, or metabolites that neutralise inhibitors of *luxS*, would enhance *luxS* expression and reduce biofilm buildup and bacterial virulence, and could be novel targets for antibacterial drug design and development. 

### 2.3. Arginine and Proline Metabolism

The *argC* gene encodes for *N*-acetyl-gamma-glutamyl-phosphate reductase. The *argC* gene plays an important role in the biosynthesis of arginine from glutamate in the amino acid metabolic pathway. It is the enzyme that catalyses the third step in the latter reaction, and without this process the production of *N*-acetylglutamate 5-semialdehyde cannot be completed from the NADP-dependent reduction of *N*-acetyl-5-glutamyl phosphate [21,22]. This reaction is therefore part of the arginine biosynthetic pathway, which is essential for some microorganisms and plants [23]. Arginine is a precursor of polyamines, and is required for the synthesis of ornithine and the polyamines putrescene, spermidine, and spermine [24]. The physiological functions of polyamines in bacteria include growth, cell wall biosynthesis, cellular differentiation, biofilm formation, stress response, proliferation, and siderophore production [25]. Upon challenge with eugenol, a virulence strategy of MRSA is to initiate biofilm production via upregulating *argC* and downregulating *luxS*. When MRSA was challenged with eugenol, the *argC* gene was shown to be upregulated by 2.0–2.5-fold. In bacteria, the *argC* protein is a monofunctional unit of 35–38 kD. In contrast, in fungi, it is bifunctional, and forms part of a mitochondrial enzyme complex (*arg5*, *6*, *arg11*, or *arg6* genes) with an N-terminal acetylglutamate kinase domain and a C-terminal AGPR domain [21,22]. In *Escherichia coli*, the enzyme—a cysteine protease—has been associated with catalytic activity around the residue region cysteine (Cys238), which is well conserved and can serve as a signature pattern [21,22]. 

Microbial secondary metabolites such as antibiotics, pigments, and toxins are produced by microorganisms as a self-defence mechanism [26]. It is thus understandably expected that challenge of MRSA with eugenol would cause upregulation of the *argC* gene, as shown in Table 2, which would in turn enhance the biosynthesis of secondary metabolites, including pigments and stress-related proteins that are normally expressed when the bacterium is subjected to physiological stress. 

## 3. Materials and Methods

### 3.1. Bacterial Strains and Growth Conditions

Methicillin-resistant *Staphylococcus aureus* (MRSA) ATCC 700698 was used as the reference strain in this study, and was kindly provided by the Department of Medical Microbiology and Parasitology, Faculty of Medicine and Health Sciences, Universiti Putra Malaysia, Serdang. The bacterial strain was cultured on Mueller–Hinton agar (MHA) for 18–24 h, after which pure, single colonies were maintained at 37 °C with shaking at 200 rpm in Mueller–Hinton broth (MHB). For growth inhibition, subinhibitory concentrations of 0.0012 mg/mL (0.00195 mM) of eugenol obtained from Sigma-Aldrich (Burlington, MA, USA) were prepared in 10% dimethyl sulfoxide (DMSO) and added immediately to the broth culture after reaching an OD_600_ of 0.7, a turbidity comparable to that of a 0.5 McFarland standard, and a bacterial count equivalent to approximately 10^6^ cfu/mL. The OD_600_ was measured using a Lambda 25 spectrophotometer (PerkinElmer, Inc., Waltham, MA, USA).

### 3.2. Total RNA Isolation and Purification

Total RNA was isolated after 1 h with and without (control) subinhibitory concentrations of eugenol, using the simple phenol method as described by Salman [15]. The quality and quantity of RNA obtained were determined using the NanoDrop 2000 Spectrophotometer (Thermo Scientific Nano Drop products, Wilmington, NC, USA) and the RNA 6000 Nano Chip-on-Chip Electrophoresis system (LOT#:QC29BK20) (Agilent Technologies, Inc., Waldbronn, Germany) with the Agilent 2100 Bioanalyser (Agilent Technologies, Inc., Santa Clara, CA, USA).

### 3.3. cDNA Synthesis, Labelling, Hybridisation, Staining, and Scanning

cDNA synthesis, labelling, purification, hybridisation, staining, and washing steps were performed according to the manufacturer’s protocol for the Agilent MRSA customised oligonucleotide microarray (Order 0304399531, Content G4102A) (Agilent Technologies Inc., Santa Clara, CA, USA).

### 3.4. Microarray Analysis

The microarray utilised for this study comprised 8 arrays with 60,000 features per array, giving rise to a total of 480,000 (60,000 × 8) features. The microarray had a specific Agilent identifier called the Agilent Microarray Design Identifier (AMADID), which was used to identify the type of array being used. The AMADID had the design ID 045256. The arrays were scanned using the DNA Microarray Scanner with SureScan High-Resolution Technology. The features extracted from the scanner were analysed using GeneSpring 12 (Agilent Technologies, Santa Clara, CA, USA) with the following parameters: *p*-value > 0.05 and fold change > 2.

### 3.5. Validation of Gene Expression by Quantitative Real-Time PCR

To determine the validity of the array data, transcriptional levels obtained using the microarray were compared with those from quantitative real-time PCR. Genes and primer sequences employed for the real-time PCR analysis are listed in Table 3 and Table 4. The housekeeping genes *arc* and *yqiL* were used as endogenous reference genes. The experiment was performed using the iCycler iQ5 Real-Time PCR Detection System (Bio-Rad Laboratories, Hercules, CA, USA) with the QuantiTect SYBR Green PCR Kit (Qiagen, Hilden, Germany). For each gene amplified, two biological replicates with three technical replicates were employed. The reaction mixtures were initially incubated for 3 min at 95.0 °C, followed by 40 cycles of 10 s at 95 °C, 30s at 55 °C, and 20 s at 72.0 °C. The PCR efficiency results were derived from standard curve slopes using the iCycler software v. 3.1 (Bio-Rad Laboratories, Hercules, CA, USA).

## 4. Conclusions

From our genomic transcriptomics data, eugenol was shown to affect targets/pathways that are involved in amino acid metabolism and protein translation, both of which are essential for development and transcriptional processes in the survival of bacteria. The functional roles and mechanisms of these target genes are present in both Gram-positive and Gram-negative bacteria. Eugenol has a high level of penetrability into the bacterial periplasm and cytoplasm, mechanistically enabled through the disruption of the cell wall and complex matrices of the cytoplasm. Although the in-depth understanding of the physicochemical properties of eugenol’s entry into MRSA is limited, the structure–activity relationship should be explored through high-throughput screening (HTS) and molecular docking to further our understanding of the molecular mechanism. The emergence of and continuous increase in bacterial resistance to antibacterial agents is currently a global challenge. Eugenol may be used in combination with currently available antibacterial agents as combination therapy to address this global pandemic of bacterial resistance. Antibiotics such as methicillin, vancomycin, and teicoplanin are known to disrupt the peptidoglycan complex, and can be used in combination with other antibacterial agents to achieve multiple-target efficiency, thereby improving their antibacterial activity. An example in this case is the study conducted on the combinatory effect of betulinaldehyde with methicillin, which reduces the minimum inhibitory concentrations (MICs) in MSSA [27]. In particular, combining eugenol with broad-spectrum inhibitors of *argC* or downstream genes, or inhibitors of protein synthesis, polyamine synthesis, alarmone ((p)ppGpp) synthesis, or the bacterial stringent response, may yield summative/synergistic effects. Interestingly, Rabin et al. [28] provided a comprehensive review of bacterial biofilm antagonists that could be administered with eugenol as a combination drug therapy. The wide array of molecules discussed as potential and proven biofilm antagonists includes bromoageliferin, oroidin, 2-aminoimidazole derivatives, benzimidazole analogues, indole–triazole–amide analogues, resveratrol and its oligomers, diphenyl disulphide, *S*-phenyl-l-cysteine sulfoxide, ajoene, brominated furanone analogues, bromopyrrole alkaloids, skyllamycins, cembranoids, synthetic *N*-acyl homoserine lactone analogues, and carolacton. With the latter extensive array of molecules in our arsenal, which could potentiate the action of eugenol, we predict that the current subinhibitory concentration of eugenol against MRSA (0.0012 mg/mL) can be significantly reduced. 

This work describes the first genome-wide transcriptional analysis of upregulated and downregulated genes in MRSA in response to eugenol, utilising a whole-genome microarray. The global transcriptomic profile of eugenol-treated MRSA shows fluctuations of genes involved in cysteine and methionine metabolism, as well as arginine and proline metabolism, as validated by qPCR. These genes that fluctuated in expression upon challenge with eugenol are important for the survival of MRSA, and show promise as novel targets for antibacterial drug design and development. Further research into the potential combinatory/synergistic effects of eugenol with *luxS* inducers and *argC* inhibitors will better facilitate our understanding of the response of both genes under stringent conditions.

## Figures and Tables

**Table 1:** List of genes expressed in response to eugenol treatment of methicillin-resistant *Staphylococcus aureus*, and their fold changes, *p*-values, and functional classifications; FC: fold change.

Probe Name	Gene ID	Gene Name	Functional Class	Protein Name	Pathway Description	Fold Change	*p*-Value	Regulation
CUST_3705_PI428639740	SAHV_0568		Carbohydrate metabolism	Hypothetical protein 3-hexulose-6-phosphate synthase	Pentose phosphate pathway	2.5	0.02	Up
CUST_5451_PI428639740	SAHV_1404		Complement	Hypothetical protein		5.4	0.003	Down
CUST_1524_PI428639740	SAHV_1631	*Obg*	Ribosome biogenesis	GTPases	GTP-binding protein	3.9	0.03	Up
CUST_1991_PI428639740	SAHV_2118	*luxS*	Amino acid metabolism; cysteine and methionine metabolism	*S*-ribosylhomocysteinase	Cysteine and methionine metabolism	2.1	0.01	Down
CUST_5450_PI428639740	SAHV_1404		Complement	Hypothetical protein		4.6	0.04	Down
CUST_2569_PI428639740	SAHV_0045		Complement	Hypothetical protein		2.2	0.02	Down
CUST_6663_PI428639740	SAHV_1949		Complement	Hypothetical protein		2.9	0.04	Down
CUST_193_PI428639740	SAHV_0183	*argC*	Amino acid metabolism; Arginine and proline metabolism	*N*-acetyl-gamma-glutamyl-phosphate reductase	Arginine and proline metabolic pathways; biosynthesis of secondary metabolites; 2-oxocarboxylic acid metabolism	2.1	0.03	Up
CUST_2081_PI428639740	SAHV_2209	*rpsK*	Genetic information processing; translation; ribosome	30S ribosomal protein S11	Ribosome	2.2	0.04	Up
CUST_4653_PI428639740	SAHV_0973		Complement	Hypothetical protein		2.7	0.02	Down
CUST_2112_PI428639740	SAHV_2219	*rplF*	Genetic information processing; translation; ribosome	50S ribosomal protein L6	Ribosome	2.4	0.05	Up
CUST_23_PI428639740	SAHV_0015	*rplI*	Genetic information processing; translation; ribosome	50S ribosomal protein L9	Ribosome	2.3	0.03	Up
CUST_3538_PI428639740	SAHV_0480		Complement	Hypothetical protein		2.4	0.01	Up

**Table 2:** Functional classes with associated upregulated and downregulated genes in control (C) vs. eugenol (E).

Functional Class(es)	Gene(s) Symbol	Upregulated	Downregulated
Translation	*rplI*, *rplF*, *rpsK*	*rplI*, *rplF*, *rpsK*	
Amino acid transportation metabolism	*luxS, argC*	*argC*	*luxS*
Translational, ribosomal, and biogenesis	*Obg*, *rplI*, *rplF*, *rpsK*	*Obg*, *rplI*, *rplF*, *rpsK*	
Arginine and proline metabolism	*argC*	*argC*	
Cysteine and methionine metabolism	*luxS*		*luxS*

**Table 3:** A comparison between microarray and qPCR results for E (eugenol) versus C (control) for the genes, *rpIF*, *rplI*, *luxS*, *argC*, and *rpsK*, using an average of 4 housekeeping genes (*glpF*, *arc*, *gmk,* and *tpiA*) for normalisation.

Gene	qPCR		Microarray		Match with Microarray
	Fold Change (Abs)	Regulation	Fold Change (Abs)	Regulation	
*rpIF*	−1.6	Down	2.3	Up	✗
*rplI*	−1.2	Down	1.2	Up	✗
*luxS*	−2.4	Down	−2.0	Down	✓
*argC*	2.5	Up	1.3	Up	✓
*rpsK*	−1.1	Down	2.2	Up	✗

**Table 4:** A comparison between microarray and qPCR results for E (eugenol) versus C (control) for the genes, *rpIF*, *rplI*, *luxS*, *argC*, and *rpsK*, using the *gmk* gene for normalisation.

Gene	qPCR		Microarray		Match with Microarray
	Fold Change (Abs)	Regulation	Fold Change (Abs)	Regulation	
*rpIF*	−1.9	Down	2.3	Up	✗
*Rpll*	−1.4	Down	1.2	Up	✗
*luxS*	−3.0	Down	−2.0	Down	✓
*argC*	2.0	Up	1.3	Up	✓
*Rpsk*	−1.3	Down	2.2	Up	✗

**Table 5:** RT-qPCR percentage of correlation with microarray data.

Treatment	Percentage Correlation with Four Housekeeping Genes (*glpF, arc, gmk, tpiA* HKG)	Percentage Correlation with One House Keeping Gene (*gmk* HKG)
E vs. C	60	53.3

## Data Availability

The data that support the findings of this study are available from Mallikarjuna Rao Pichika, but restrictions apply to the availability of these data, which were used under license for the current study, and are therefore not publicly available. However, data are available from the authors upon reasonable request, and with the permission of Mallikarjuna Rao Pichika.

## Sample Availability

Samples of the compounds are available from the authors.
